# A DAO1-Mediated Circuit Controls Auxin and Jasmonate Crosstalk Robustness during Adventitious Root Initiation in *Arabidopsis*

**DOI:** 10.3390/ijms20184428

**Published:** 2019-09-09

**Authors:** Abdellah Lakehal, Asma Dob, Ondřej Novák, Catherine Bellini

**Affiliations:** 1 Umeå Plant Science Centre, Department of Plant Physiology, Umeå University, SE-90736 Umeå, Sweden; 2 Laboratory of Growth Regulators, Faculty of Science, Palacký University and Institute of Experimental Botany, The Czech Academy of Sciences, 78371 Olomouc, Czech Republic; 3 Umeå Plant Science Centre, Department of Forest Genetics and Physiology, Swedish Agriculture University, SE-90183 Umea, Sweden; 4 Institut Jean-Pierre Bourgin, INRA, AgroParisTech, CNRS, Université Paris-Saclay, FR-78000 Versailles, France

**Keywords:** organogenesis, adventitious roots, jasmonates, auxin, auxin oxidation

## Abstract

Adventitious rooting is a post-embryonic developmental program governed by a multitude of endogenous and environmental cues. Auxin, along with other phytohormones, integrates and translates these cues into precise molecular signatures to provide a coherent developmental output. Auxin signaling guides every step of adventitious root (AR) development from the early event of cell reprogramming and identity transitions until emergence. We have previously shown that auxin signaling controls the early events of AR initiation (ARI) by modulating the homeostasis of the negative regulator jasmonate (JA). Although considerable knowledge has been acquired about the role of auxin and JA in ARI, the genetic components acting downstream of JA signaling and the mechanistic basis controlling the interaction between these two hormones are not well understood. Here we provide evidence that *COI1*-dependent JA signaling controls the expression of *DAO1* and its closely related paralog *DAO2*. In addition, we show that the *dao1-1* loss of function mutant produces more ARs than the wild type, probably due to its deficiency in accumulating JA and its bioactive metabolite JA-Ile. Together, our data indicate that *DAO1* controls a sensitive feedback circuit that stabilizes the auxin and JA crosstalk during ARI.

## 1. Introduction

Adventitious rooting is a post-embryonic developmental program enabling new roots to arise and branch out from the aboveground plant organs. The ability of plant species to reprogram their differentiated cells into new meristematic-like cells requires precise molecular signatures. These signatures can be generated by an intrinsic developmental cue or by a multitude of environmental ones [1,2,3]. The ability of plants to perceive and translate these inductive cues is an evolutionary fitness trait, providing them with the capacity to regenerate and clonally propagate as well as form extra roots whenever needed. How plants sense and integrate different inductive cues to trigger cell-identity transition programs leading to adventitious root initiation (ARI) is poorly understood. Nevertheless, it is evident that, along with several signaling molecules, phytohormones play a prominent role in the integration of these cues to define suitable cell-fate decisions [4].

The phytohormone auxin is one of the central integrators of the inductive cues during ARI. Early stages of ARI, including cell reprogramming and cell identity transitions, require generation of an auxin gradient in specific cell types. These gradients are generated by the action of multiple and coordinated mechanisms that include polar auxin transport (PAT) [5], local de novo biosynthesis, and homeostasis [6,7]. Auxin homeostasis is also controlled by multiple mechanisms that include reversible (transient) conjugation and irreversible conjugation, as well as oxidation [8,9].

Irreversible conjugation of indole-3-acetic acid (IAA) into amino acids is mainly catalyzed by GRETCHEN HAGEN3 (GH3) acyl-amido synthetases [10]. This type of conjugation is thought to serve as a regulator of the local auxin availability in response to environmental cues [11,12]. IAA oxidation is the main route of auxin catabolism in *Arabidopsis* [13]. The first putative auxin oxidase was found in apple (*Malus domestica*), and was named *ADVENTITIOUS ROOTING RELATED OXYGENASE1* (*ARRO-1*) [14]. *ARRO-1* was highly upregulated during indole-3-butyric acid (IBA)- or IAA-induced adventitious rooting from apple stem cuttings [14], suggesting that this gene plays an important role in regulating auxin availability during stem cutting-derived AR formation in apple and probably also in other species. The enzyme catalyzing the conversion of free IAA into 2-oxindole-3-acetic acid (oxIAA) was first cloned in rice (*Oryza sativa*), and was named *DIOXYGENASE FOR AUXIN OXIDATION* (*DAO*) [15]. Os*DAO* is an evolutionarily conserved gene that belongs to the 2-oxoglutarate–dependent Fe (II) dioxygenase gene family. The Os*DAO* gene controls anther dehiscence and pollen fertility via auxin-mediated JA biosynthesis inhibition in rice [15]. The anthers of the Os*dao* loss of function mutant accumulated less JA than the wild type, due to the downregulation of several key genes in the JA biosynthesis pathway [15]. These data suggest that the Os*DAO* gene plays a crucial role in auxin and JA crosstalk during anther dehiscence and possibly in other developmental programs. In *Arabidopsis* (*Arabidopsis thaliana*), At*DAO* genes have been found in a phylogenetic screen for homologs of the rice Os*DAO* [16,17]. The *Arabidopsis* genome contains two closely related paralogs—*DAO1* and *DAO2*. A labeled-IAA feeding experiment in *Arabidopsis* seedlings showed that the *dao1-1* loss of function mutant produced a small amount of oxIAA catabolite, but the mutant retained the same amount of free IAA as the wild type [16,17]. Further analysis of the IAA–amino acid conjugates revealed that *dao1-1* accumulates a large amount of the conjugates indole-3-acetyl-L-aspartic acid (IAAsp) and indole-3-acetyl glutamic acid (IAGlu) [16,17]. These data indicate that the amount of free IAA in *dao1-1* is maintained at the wild-type level by the GH3-mediated compensatory pathway [16,17,18]. The DAO2 protein was also shown to oxidize IAA into oxIAA in vitro [16], suggesting that the two genes act redundantly to control IAA levels in planta. Interestingly, it has been shown that *DAO1* expression was only mildly induced in response to exogenously applied IAA, suggesting other transcriptional regulation mechanisms. How *DAO*s are transcriptionally regulated to control IAA degradation is not yet known.

The oxylipin-derived phytohormone jasmonate (JA) is known to counteract or cooperate with auxin to regulate a number of developmental programs including ARI, but the mechanistic basis of these interactions is not well understood. Nevertheless, physiological studies have reported that exogenously applied JA or its derivative methyl-jasmonate (MeJA) enhances IAA biosynthesis in a *CORONATINE INSENSTIVE1* (*COI1*)-dependent manner during lateral root (LR) formation [19]. *COI1* is the nuclear receptor of the bioactive form jasmonoyl-L-isoleucine (JA-Ile) [20]. Furthermore, MeJA modulates the subcellular localization of the auxin influx carrier PIN-FORMED2 in the *Arabidopsis* root in a COI1-dependent manner, suggesting that JA signaling controls the PAT [21]. We have previously shown that *AUXIN RESPONSE FACTOR 6* (*ARF6*) and *ARF8*-mediated auxin signaling inhibit JA accumulation by enhancing its inactivation into amino acid conjugates in *Arabidopsis* hypocotyls during ARI [22]. Another prime example of the complexity of IAA and JA crosstalk is reflected in their role during flower development. Depending on the developmental stage, auxin signaling may enhance or inhibit JA biosynthesis to control the development of the male reproductive organs [23,24]. These examples indicate that the interaction between auxin and JA is complex and requires further research to unravel the key components that modulate and stabilize these interactions.

Here we show that JA controls the transcription of *DAO1* and *DAO2* in a dose- and time-dependent manner. This regulation requires a functional COI1-dependent signaling pathway. By creating a *dao1–1dao2C* double mutant using CRISPR-Cas9 technology, we showed that DAO1 is the major player in auxin degradation during ARI. In addition, we showed that the enhanced AR phenotype in *dao1-1* loss of function is probably due to a reduced amount of free JA and its bioactive metabolite JA-Ile. In conclusion, we propose that DAO1 is one of the key components of IAA–JA crosstalk during ARI.

## 2. Results

### 2.1. Jasmonate Induces the Expression of DAO1 and DAO2 in a COI1-Dependent Manner

We have previously shown that ARI is controlled by a complex crosstalk involving auxin signaling and JA homeostasis [22]. To identify novel players acting downstream of JA with a potential role in ARI, we screened the publicly available JA-related transcriptomic datasets. Among several candidates, we found the recently characterized *DAO1* and its closely related paralog *DAO2* [16,17,18]. These two genes were differentially expressed in several JA-related transcriptome profiling experiments [25,26,27] and seem to be specifically induced by exogenously applied JA, as indicated by the *Arabidopsis* eFP browser [28] (Figure S1). Because JA is one of the primary mediators of mechanical wounding, we also searched in the recent publicly available wounding-related transcriptomic datasets [29], and interestingly we found that wounding rapidly induced *DAO1* (i.e., within 10 min), peaking within 30 min and with sustained upregulation for 12 h at the wounding site of an *Arabidopsis* leaf explant [29]. These data suggest that *DAO* genes play an important role at the crossroads of auxin and JA interaction during JA and wounding-mediated ARI.

**Figure 1:**
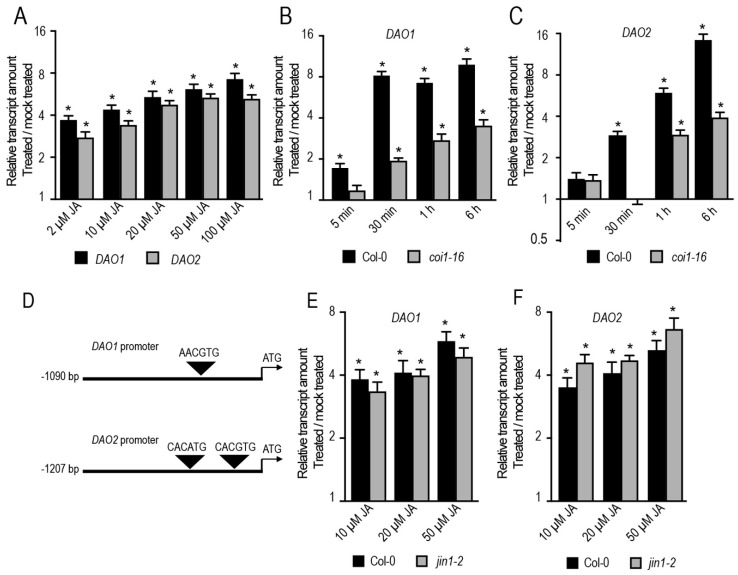
Jasmonate (JA) controls the expression of *DAO1* and *DAO2.* (**A**) Relative transcript amount of *DAO1* and *DAO2* quantified by qRT-PCR. mRNAs were extracted from six-day-old wild-type seedlings treated for 1 h either with different doses of JA or mock solution. The gene expression values are relative to the mock-treated control, for which the value was arbitrarily set to 1. The scale in the Y axis is indicated as a log2 unit. Error bars indicate ± SD obtained from three technical replicates. (**B**,**C**) Relative transcript amount of *DAO1* and *DAO2* quantified by qRT-PCR. mRNAs were extracted from six-day-old wild-type or *coi1-16* mutant seedlings treated with 50 μM JA or mock solution at different time points. The gene expression values are relative to the mock-treated control, for which the value was arbitrarily set to 1. Error bars indicate ± SD obtained from three technical replicates. (**D**) Representative scheme of the location of G-box or G-box-like *cis* regulatory elements on the *DAO1* and *DAO2* promoters. (**E**,**F**) Relative transcript amount of *DAO1* and *DAO2* quantified by qRT-PCR. mRNAs were extracted from six-day-old wild-type or *jin1-2* mutant seedlings treated for 1 h with different JA doses or mock solutions. The gene expression values are relative to the mock-treated control, for which the value was set to 1. Error bars indicate ± SD obtained from three technical replicates. A *t*-test indicates that the values indicated by an asterisk are significantly different from their mock counterpart (*p* < 0.01; *n* = 3). All wild-type and mutant seedlings were grown for five days under long-day conditions (16 h light/8 h dark), then they were acclimated overnight in liquid media before any treatment. All the experiments were repeated with another independent biological replicate and gave similar results.

To confirm that JA induces *DAO1* and *DAO2*, we first quantified the relative transcript amount, using qRT-PCR, of these two genes in wild-type *Arabidopsis* seedlings (Col-0 ecotype) treated for 1 h with different doses of JA. Both *DAO1* and *DAO2* were upregulated in a dose-dependent manner (Figure 1A), consistent with the published transcriptomic datasets [28]. Next, we investigated whether JA-mediated *DAO1* and *DAO2* induction requires a functional COI1 receptor. We quantified the relative transcript amount of *DAO1* and *DAO2* in both the wild type and the weak-allele mutant *coi1-16* treated either with 50 μM JA or mock solution. In the wild type, the *DAO1* transcript amount was rapidly upregulated within 5 min, reaching a maximum within 30 min, whereas it was not induced in *coi1-16* 5 min or 30 min after treatment. It was only slightly induced within 1 h of treatment (Figure 1B). Notably, similar results were obtained for *DAO2*, except that its induction in the wild type was within 30 min and upregulation was sustained for 6 h after treatment (Figure 1C). These data indicate that JA induces the expression of *DAO1* and *DAO2* in a dose/time-dependent manner and it requires functional COI1-dependent JA signaling.

To test further whether JA-mediated *DAO1* and *DAO2* expression requires the basic helix–loop–helix *JASMONATE INSENSTIVE1* (*JIN1/MYC2*) transcription factor which is the master regulator of the JA signaling pathway, we first manually scanned the promoter sequences of *DAO1* and *DAO2*, searching for G-box or G-box-like *cis* regulatory elements, which are the preferred binding sites of MYC2 [30]. Interestingly, we found one G-box-like (AACGTG) motif within 396 base pairs (bp) upstream of the translation start codon of *DAO1* (Figure 1D). We also found two canonical G-box (CACGTG, CACATG) motifs, respectively, within 356 bp and 556 bp upstream of the translation start codon of *DAO2* (Figure 1D). Although direct experimental evidence such as ChIP and/or EMSA would be required, these data suggest that MYC2 may directly regulate the expression of *DAO1* and *DAO2*. We next quantified the relative transcript amount of these two genes in the wild type and the *jin1-2/myc2* loss of function mutant treated with different JA concentrations for one hour. As shown in Figure 1E,F, JA induced the expression of *DAO1* and *DAO2* in the wild type and *jin1-2* loss of function mutant in a similar manner. These data suggest either that JA acts independently of MYC2 or that MYC2 acts redundantly with MYC3 and MYC4 to control the expression of these two genes.

### 2.2. JA Induces the Expression of DAO1 and DAO2 Independently of TIR1/AFB-Dependent Auxin Signaling

**Figure 2:**
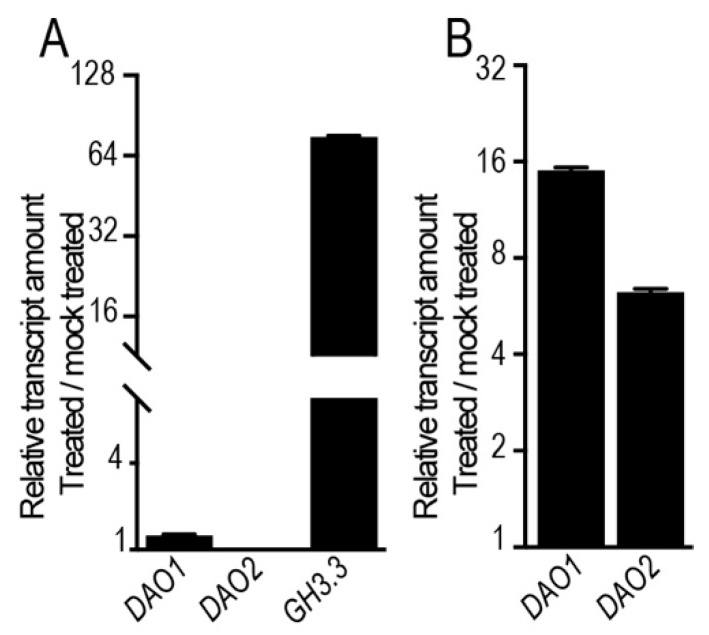
JA controls the expression of *DAO1* and *DAO2* independently of TIR1/AFB-dependent auxin signaling. (**A**) Relative transcript amount of *DAO1*, *DAO2*, and *GH3.3* quantified by qRT-PCR. mRNAs were extracted from six-day-old wild-type seedlings treated for 1 h with 10 μM indole-3-acetic acid (IAA) or mock solutions. The gene expression values are relative to the mock-treated control, for which the value was set to 1. (**B**) Relative transcript amount of *DAO1* and *DAO2* quantified by qRT-PCR. mRNAs were extracted from six-day-old wild-type seedlings pre-treated for 90 min with 10 μM auxinole, then they were co-treated with 50 μM JA and 10 μM auxinole for 1 h. The gene expression values are relative to the mock-treated control, for which the value was set to 1. The scale in the Y axis is indicated as a log2 unit. Error bars indicate ± SEM obtained from three technical replicates. Wild-type seedlings were grown for five days under long-day conditions (16 h light/8 h dark), then they were acclimated overnight in liquid media before any treatment. All the experiments were repeated at least twice and the biological replicates gave the same results.

JA promotes IAA biosynthesis by inducing the expression of several key genes in the tryptophan-dependent pathway; these include *ANTHRANILATE SYNTHASE ALPHA 1* (*ASA1*), *YUCCA2* (*YUC2*), *YUC4*, *YUC8*, and *YUC9* [19,31,32]. In addition, combined mathematical modeling and experimental approaches reveal that exogenously applied IAA slightly induces the expression of *DAO1* [18]. These considerations raise the possibility that JA may induce the expression of *DAO1* and *DAO2* upstream of the auxin signaling machinery. To verify this, we first quantified the relative transcript amount of *DAO1* and *DAO2* genes in wild-type seedlings treated for 1 h either with 10 μM IAA or mock solution. Under the conditions imposed here, the expression of these two genes was not (or only slightly) induced by IAA, which, in contrast, greatly induced (more than 70-fold) the expression of the known auxin-responsive gene *GH3.3* (Figure 2A). Next, taking a pharmacological approach, we checked whether JA controls the expression of these two genes independently of TIR1/AFB-dependent auxin signaling. We first pre-treated wild-type seedlings with 10 μM auxinole to block the auxin perception machinery.

Auxinole is a potent auxin antagonist which binds to the TIR1/AFB receptors and consequently eliminates their function [33]. We co-treated the same seedlings with 50 μM JA and 10 μM auxinole. Interestingly, JA induced the expression of *DAO1* and *DAO2* even in the presence of auxinole (Figure 2B), indicating that JA controls the expression of these two genes independently of the TIR1/AFB-dependent auxin signaling pathway. This conclusion is also supported by the fact that JA induces *DAO1* rapidly, within 5 min of treatment (Figure 1B).

### 2.3. DAO1, but not DAO2, Controls Adventitious Root Initiation

**Figure 3:**
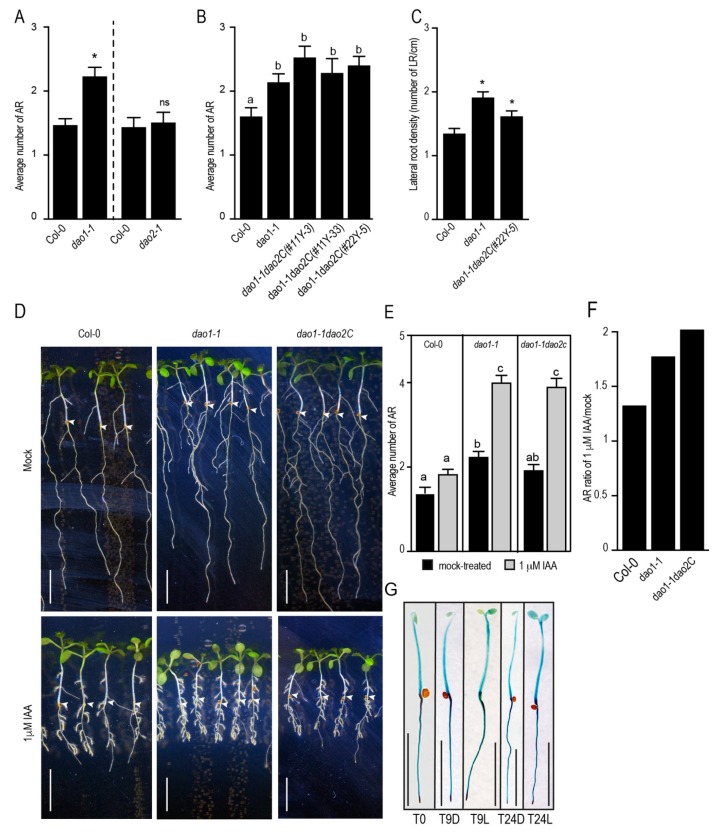
*DAO1*, but not *DAO2*, plays a major role in IAA oxidation during ARI. (**A**,**B**) Average number of ARs in *dao* mutants. Seedlings were grown in AR phenotyping conditions. (**A**) A *t*-test indicates that the *dao1-1* mutant exhibits significantly more ARs than the wild type (inidicated by an asterisk). Error bars indicate ± SEM (*n* > 30; *p* < 0.001). (**B**) One-way ANOVA combined with Tukey’s multiple comparison post-hoc test indicates that the *dao1-1* single and *dao1–1dao2C* double mutants exhibit significantly more ARs compared to the wild type. The values indicated by different letters are significantly different from each others. Error bars indicate ± SEM (*n* > 20; *p* < 0.001). (**C**) Density of lateral roots (LRs) (i.e., the number of LRs per cm of primary root) in *dao* mutants grown in AR phenotyping conditions. One-way ANOVA combined with Dunnett’s multiple comparison post-hoc test indicated that the LR density was significantly affected in *dao1-1* single and *dao1–1dao2C* double mutants (inidicated by an asterisk). Error bars indicate ± SEM (*n* > 20; *p* < 0.001). (**D**) Wild-type and *dao* mutant seedlings were grown in the dark until their hypocotyls reached 6 mm long, when they were transferred to fresh medium containing either mock solution or 1 μM IAA. The seedlings were kept for seven more days under long-day conditions to induce ARs. Arrow heads indicate hypocotyl–root junction. (**E**) Average number of ARs in the wild type and *dao* mutants in response to IAA grown as in (**D**). One-way ANOVA combined with Tukey’s multiple comparison post-hoc test indicates that the *dao1-1* single and *dao1–1dao2C* double mutant produce significantly more ARs than the wild type. The values indicated by different letters are significantly different from each others. Error bars indicate ± SEM (*n* > 30; *p* < 0.001 (**F**) Ratio of AR number from IAA-treated/mock-treated seedlings. One-way ANOVA combined with Tukey’s multiple comparison post-hoc test indicates that the *dao1-1* single and *dao1–1dao2C* double mutant produce significantly more ARs than the wild type. Error bars indicate ± SEM (*n* > 30; *p* < 0.001). (**G**) Spatiotemporal activity and dynamics of *DAO1* promoter. Seedlings expressing the *pDAO1:GUS* construct were grown in the dark until their hypocotyls were 6 mm long (T0), 9 h (T9L), and 24 h (T24L) after their transfer to the light and their respective controls, which were kept in the dark for 9 h (T9D) and 24 h (T24D). The seedlings were stained for 2 h. (**D**–**G**) All scale bars represent 6 mm.

To test whether *DAO1* and *DAO2* have any biological relevance in terms of adventitious rooting, we first counted the number of ARs in *dao1-1* and *dao2-1* loss of function mutants. Notably, only the *dao1-1* mutant exhibited a slight increase in AR number compared to the wild type, whereas the *dao2-1* mutant retained a wild-type phenotype (Figure 3A). It has been suggested that *DAO1* and *DAO2* probably act redundantly to control IAA degradation [16].

To verify the genetic interaction between these two genes during ARI, we generated a double mutant *dao1–1dao2C* by deleting a large DNA fragment from the first exon and part of the second exon including the intron from the *DAO2* gene in the *dao1-1* mutant background using CRISPR-Cas9 technology (Figure S2A). The large deletion of approximately 500 bp in the *DAO2* gene probably creates an aberrant and unfunctional mRNA (Figure S2B). We analyzed the AR phenotype of two independent double mutant *dao1–1dao2C* plants and found that they exhibited the same number of ARs as the *dao1-1* single mutant (Figure 3B). We checked under our growth conditions the LR phenotype of *dao1-1* and *dao1–1dao2C* mutants. The LR density was significantly higher in *dao1-1* and *dao1–1dao2C* mutants than in the wild type (Figure 3C). This is in agreement with previous reports [15,17]. Furthermore, we tested the responsiveness of *dao1-1* and *dao1–1dao2C* mutants to exogenously applied IAA. One μM IAA was not sufficient to significantly stimulate AR production in the wild type, whereas it dramatically stimulated the formation of AR in the single *dao1-1* and double *dao1–1dao2C* mutants (Figure 3D,E). Notably, *dao1-1* and *dao1–1dao2C* exhibited the same response to exogenously applied IAA, as shown in Figure 3F. These data indicate that *dao1-1* and *dao1–1dao2C* exhibit the same hypersensitivity to exogenously applied IAA. Together, these results suggest that, during ARI, DAO1 is the major player controlling auxin homeostasis, while DAO2 plays a minor role. Therefore, we subsequently focused our efforts on the characterization of the role of *DAO1* in ARI.

We assessed the spatiotemporal activity of *DAO1* promoter during the early stages of ARI using the pDAO1:GUS (ß-glucuronidase) transcriptional fusion line [17]. As shown in Figure 3G, *DAO1* promoter was ubiquitously active in the whole etiolated seedlings. We did not observe any effect of light on *DAO1* promoter activity (Figure 3G). These data indicate that *DAO1* probably controls IAA in the whole seedling both in the dark and in the light.

### 2.4. The dao1-1 Mutant Produces Less JA and JA-Ile in the Etiolated Hypocotyls

**Figure 4:**
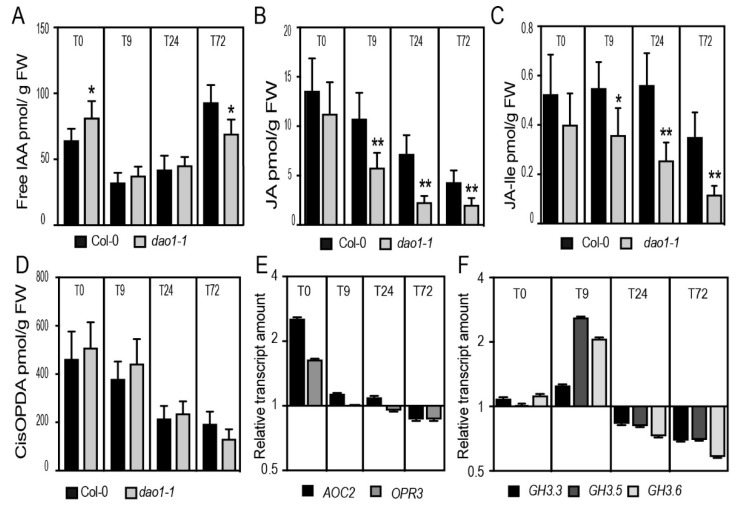
The AR phenotype of the *dao1-1* mutant is probably linked to its deficiency in JA and JA-Ile. (**A**–**D**) Endogenous hormone contents. (**A**) Free IAA, (**B**) free JA, (**C**) JA-Ile, and (**D**) *cis*-OPDA were quantified in the hypocotyls of wild-type and *dao1-1* mutant seedlings grown in the dark until the hypocotyl reached 6 mm long (T0) and after their transfer to the light for 9 h (T9), 24 h (T24) and 72 h (T72). Asterisks indicate a statistically significant difference in the mutant lines versus the wild type in an ANOVA analysis (* and **correspond to *p*-values of 0.05 > *p* > 0.01, 0.01 > *p* > 0.001). Error bars indicate ± SD of six biological replicates. (**E**) Relative transcript amount of two key genes in the JA biosynthesis, *AOC2 and OPR3*, as well as (**F**) *GH3.3*, *GH3.5*, *and GH3.6* quantified by qRT-PCR. mRNA was extracted from hypocotyls of wild-type and *dao1-1* mutant seedlings grown in AR phenotyping conditions as indicated above. The gene expression values are relative to the wild type, for which the value was set to 1. The scale in the Y axis is indicated as a log2 unit Error bars indicate ± SEM obtained from three technical replicates.

To gain an insight into the role of *DAO1* in ARI, we performed hormone profiling in the etiolated hypocotyls of the wild type and *dao1-1* mutant during the early stages of ARI. We first confirmed that *dao1-1* hypocotyls accumulated less oxIAA but retained the same amount of free IAA as the wild type [17] (Figure 4A and Figure S3A). This is probably due to the upregulation of the irreversible IAA conjugation to IAGlu and IAAsp as reported by [16,17] and shown in Figure S3B,C. Notably, we observed that *dao1-1* accumulated slightly more, yet statistically significant, free IAA compared to the wild type at T0 (dark conditions) (Figure 4A). This observation is important and it reflects the complexity of the light and IAA homeostasis crosstalk. Although we are aware that *dao1-1* possibly accumulates more IAA in a cell type-specific manner, we propose that the phenotype of *dao1-1* cannot be exclusively explained by the accumulation of IAA. We have previously shown that auxin signaling promotes ARI by enhancing the conjugation of the negative regulator JA into amino acids. The conjugation process significantly contributes to the depletion of the JA pool in the hypocotyls [22]. In addition, the fertility defect in the Os*dao* loss of function mutant in rice was found to be correlated to JA deficiency. This deficiency was due to auxin-mediated downregulation of JA biosynthesis genes [15]. These considerations prompted us to hypothesize that the increase in AR number in the *dao1-1* mutant was due rather to a reduced amount of JA and JA-Ile. To verify this hypothesis, we first quantified the amount of JA and JA-Ile in the wild type and in the *dao1-1* mutant during the early stages of ARI. As expected, the *dao1-1* mutant accumulated significantly less JA and JA-Ile as compared to the wild type across all the time points tested with the exception of T0 (Figure 4B,C). To verify whether this reduction is due to a downregulation of the biosynthesis or an increase of the conjugation, we also quantified the amount of *cis*-12-oxo-phytodienoic acid (*cis*-OPDA), which is a precursor of JA, in the wild type and the *dao1-1* mutant, and observed no difference between the two (Figure 4D). Similarly, the expression of the *ALLENE OXIDE CYCLASE2* (*AOC2*) and *OXOPHYTODIENOATE-REDUCTASE3* (*OPR3*) genes, which are key genes in JA biosynthesis, was not affected in *dao1-1* (Figure 4E). We observed a slight upregulation of these two genes in *dao1-1* at T0 but the amount of *cis*-OPDA was not affected at this time point. These data suggest that the JA biosynthesis pathway is not affected in the *dao1-1* mutant and the reduction in JA and JA-Ile is possibly due to an increase in conjugation. Because we have previously reported that GH3.3, GH3.5, and GH3.6 enzymes conjugate JA into amino acid conjugate leading to JA depletion in the hypocotyls [22], we quantified the relative transcript amount by qRT-PCR of *GH3.3*, *GH3.5*, and *GH3.6*. Interestingly, we found that the expression of *GH3.5* and *GH3.6* was upregulated in *dao1-1* as compared to the wild type only at T9 (Figure 4F).

Notably, JA and JA-Ile dramatically decreased in the hypocotyls of both wild type and *dao1-1* when the etiolated seedlings were shifted from dark to light (Figure 4B–D), which is in line with our previous reports [22].

Although we cannot exclude the upregulation of other catabolic pathways responsible for the degradation of JA and JA-Ile in the *dao1-1* background, the upregulation of *GH3.5* and *GH3.6* may partly explain the reduction of JA and JA-Ile in *dao1-1* and, consequently, its AR phenotype.

## 3. Discussion

**Figure 5:**
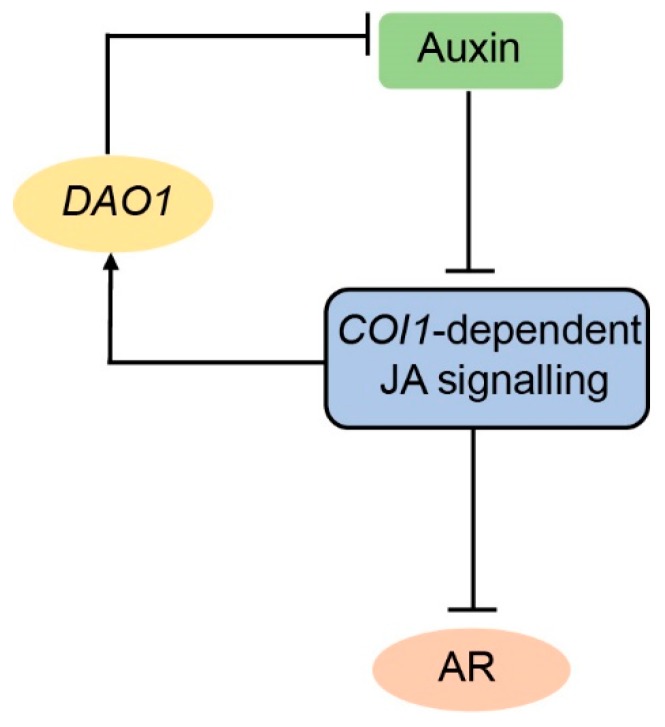
*DAO1* controls a feedback circuit to stabilize IAA–JA crosstalk during ARI. Auxin promotes ARI by modulating the homeostasis of the negative regulator JA. COI1-dependent JA signaling induces the expression of *DAO1,* which in turn controls the thresholds of IAA by irreversible degradation. Arrows indicate positive regulation, whereas dashes indicate negative regulation.

ARI is a post-embryonic developmental program governed by a number of hormone signaling pathways [4] that interact and regulate each other at different levels to provide rapid molecular signatures in response to dynamic inductive cues. Genetic and biochemical approaches showed that ARI in the etiolated *Arabidopsis* hypocotyl is controlled by a complex hormonal crosstalk involving auxin and JA signaling pathways [22,34,35]. Auxin signaling acts through three transcription factors from the *AUXIN RESPONSE FACTOR* (*ARF*) gene family. *ARF6* and *ARF8* are positive regulators, whereas *ARF17* is a negative regulator of ARI. The three ARFs control the expression of GH3.3, GH3.5, and GH3.6 enzymes. These enzymes catalyze the conjugation of free IAA and free JA into amino acids to maintain their homeostasis. In the etiolated hypocotyl, their induction by ARF6 and ARF8 causes a depletion of the JA pool and the subsequent induction of ARI [22]. We showed that JA inhibits ARI through the master regulator MYC2 transcription factor in a COI1-dependent manner [22] (Figure 5). This is in line with physiological approaches showing that continuous JA or MeJA applications inhibit AR formation in *Bupleurum kaoi* [36], *Petunia hybrida* leafy cuttings [37], and *Arabidopsis* leaf explants [29].

By searching the publicly available JA-related transcriptomic datasets, we identified novel components from the auxin catabolism machinery acting downstream of JA. We found that the enzymes DAO1 and DAO2 are consistently induced by exogenously applied JA. We experimentally confirmed this observation and found that these two genes are, indeed, transcriptionally regulated by COI1-dependent JA signaling and, possibly, are downstream targets of MYC transcription factors. A number of reports indicated that MYC2, the master regulator, acts redundantly with MYC3, MYC4 [38], and also with MYC5 [39] to control JA-mediated transcriptional cascades. These data may explain the partial responsiveness of *jin1-2* loss of function to JA in terms of *DAO1* and *DAO2* induction. Nevertheless, further research and more direct evidence, such as ChIP and/or EMSA experiments, are required to verify whether these two genes are direct targets of MYC2.

Interestingly, transcriptome analysis showed that *DAO1* was rapidly upregulated within 10 min at the wounding site of *Arabidopsis* leaf explants during ARI [29]. These data are in agreement with the fact that JA rapidly induced *DAO1* expression within 5 min of treatment. Besides inducing *DAO1* expression, wounding has also been shown to enhance both the abundance of the auxin transporter ATP-BINDING CASSETTE B19 and the IAA biosynthesis genes, leading to a local increase in free IAA, which has been linked to regeneration mechanisms and ARI [5,6,29,40]. However, what would be the significance of *DAO1* induction if it was proposed that wounding promotes regeneration and de novo ARI processes by enhancing both auxin biosynthesis and transport? One of the possible explanations is that DAO1 acts as a rapid modulator of the spatiotemporal availability of free IAA upon mechanical wounding. Thus, DAO1 would be involved in establishing the precise auxin gradients by irreversibly degrading the excess IAA generated because of either an auxin transport jam or enhanced biosynthesis. In this scenario, JA would have, in fact, a dual role in generating and maintaining the IAA gradients, first enhancing IAA production, and second controlling the threshold of this production through DAO1-mediated degradation. How these two contradictory processes are regulated requires further investigation. Further research is also needed to identify and uncouple the direct mediator(s) of the mechanical wounding involved in the control of ARI. Besides JA, ethylene and cytosolic calcium dynamics also rapidly mediate mechanical wounding signals [41] and thereby may also have a significant role in the ARI process.

Under the conditions in our study, the double mutant *dao1–1dao2C* had the same AR phenotype as the *dao1-1* single mutant, suggesting that DAO1 is the major player in auxin degradation during ARI. Using a hormone profiling approach, we found that *dao1-1* hypocotyls accumulate slightly more, yet statistically significant, IAA in dark conditions, suggesting a possible role of light in auxin homeostasis. Although the role of light in auxin biosynthesis and transport is complex and involves multiple pathways [42], we suggest that light may also control the amount of free IAA by modulating conjugation or degradation rates. Notably, the expression of several *GH3* genes is regulated by light in a *PHYTOCHROME A* (*PHYA*)- and (*PHYB*)-dependent manner [42]. Interestingly, *PHYTOCHROME INTERACTING FACTORS 4* may directly control the expression of *GH3.3*, *GH3.5*, *GH3.6*, and *GH3.17* as indicated by a ChIP experiment [43]. Under the conditions studied here, we did not see any significant difference between *dao1-1* and the wild type in terms of *GH3.3*, *GH3.5*, and *GH3.6* gene expression at T0; thus, it is unlikely that these genes are responsible for free IAA accumulation in *dao1-1*. It would be interesting to check the expression of other *GH3* genes as well as key players in auxin-homoeostasis between *dao1-1* and the wild type at T0 in order to explain the difference observed in free IAA between them at this time point.

The fact that *dao1-1* accumulates a similar amount of free IAA as the wild type (even if both are supplied with exogenous IAA as described by [17]) raises obvious questions. What is the physiological trigger of ARI in *dao1-1*? What are the physiological bases triggering hypersensitivity in terms of AR number of this mutant when treated with exogenous IAA? Although we cannot rule out the possibility that *dao1-1* may accumulate free IAA in a cell type-specific manner, as assumed by [18], we suggest that the AR phenotype is linked to JA deficiency. This suggestion is supported by the fact that *dao1-1* accumulates less JA and JA-Ile at all time points tested (T9, T24, and T72), with the exception of T0. This reduction could be due to an increase in the GH3-mediated conjugation because we observed that *GH3.5* and *GH3.6* were upregulated *in dao1-1* at 9 h after transfer to the light. This hypothesis is supported by our previous reports showing that AR number correlates with the expression levels of *GH3.3*, *GH3.5*, and *GH3.6* genes [22,44,45].

The fertility defect in the Os*dao* rice loss of function mutant was also linked to JA and JA-Ile deficiency due to the downregulation of JA biosynthesis [15]. Under the conditions in our study, the *dao1-1* mutant is unlikely to be affected in JA biosynthesis since the amount of the JA precursor *cis*-OPDA and the expression of key genes in the JA biosynthesis pathway *OPR3* and *AOC2* are not affected in this mutant.

Considering the fact that JA is a negative regulator of intact hypocotyl-derived AR, we reason that JA-induced *DAO1* has a significant biological relevance in the IAA–JA interaction by controlling the timing of the negative effect of auxin signaling on JA pools, because a minimum amount of JA and JA-Ile is needed for a proper seedling establishment and response to the environment. In fact, JA-induced *DAO1* probably attenuates the continuous negative effect of auxin signaling on JA and JA-Ile pools by either modulating or terminating the auxin signaling through degrading free IAA (Figure 5). This type of feedback loop provides sensitive timing and positional information for ARI. Whether JA-induced *DAO1* contributes in other developmental contexts awaits further investigation.

## 4. Materials and Methods

### 4.1. Plant Material

*Arabidopsis thaliana* ecotype Columbia (Col-0) was used as the wild type and background for all mutants and transgenic lines. The *dao1-1* (SALK_093162), pDAO1:GUS [17], and *dao2-1* (Salk_205223) seeds were a gift from Professor Karin Ljung. The *jin1*-2 [46] and *coi1-16* [47] seeds were a gift from Laurens Pauwels.

### 4.2. Growth Conditions, Adventitious and Lateral Root Phenotyping

All phenotyping experiments were performed in the adventitious rooting growth conditions as previously described by [7,22,34]. Mainly, after sterilization, seeds were sown in Petri dishes on a medium as described by [48] with some modifications. The medium contained 70 mM H_3_BO_3_, 0.5 mM CuSO_4_, 0.2 mM NaMoO_4_, 0.01 mM CoC1_2_, 14 mM MnC1_2_, 1mM ZnSO_4_, 10 mM NaC1, 5 mM KNO_3_, 2.5 mM KH_2_PO_4_, 2 mM MgSO_4_, 2 mM Ca(NO_3_) 2, 0.005% (*w*/*v*) ammoniacal iron (III) citrate, 3.5 mM 2-(N-morpholino) ethanesulfonic acid (MES), 1% (*w*/*v*) saccharose, and 0.7% (*w*/*v*) plant agar (Duchefa Biochemie, Haarlem, The Netherlands), pH 5.9. The dishes were kept at 4 °C for 48 h. Seed germination was induced by 8 h of light (130–135 µmol/m^2^/s). The seedlings were etiolated in the dark until the hypocotyls reached approximatively 6 mm long, then they were moved to long-day conditions (16 h light at 22 °C and 8 h dark at 17 °C, 130–135 µmol/m^2^/s and 65% relative humidity). The number of primordia as well as the number of emerged ARs were scored under a binocular stereomicroscope seven days after moving the seedlings to the light. The number of lateral roots was scored from scanned plates the same day. The primary root length was measured using ImageJ software (version number, manufacturer, city, state abbreviation, country) [49]. The lateral root density was calculated as a ratio between lateral root number and primary root length. At least 30 seedlings were used for each measurement. Three independent biological replicates were included in each experiment. For auxin sensitivity assay, seedlings were etiolated in the dark until their hypocotyl reached 6 mm long, then they were transferred to the same medium supplemented either with 1 μM IAA (Duchefa Biochemie, I0901) or mock solution.

### 4.3. CRISPR-Cas9 Vector Construction, Plant Transformation, and Genotyping

To generate the *dao1–1dao2C* double mutant, the *DAO2* gene was CRISPRed in a *dao1-1* loss of function mutant background. Two guide RNAs (DAO2_gRNA_F GTCATTCCAACAATAGACTTGG and DAO2_gRNA_R TTAGCGGAGAGCTACGGAGTGG) were designed to target the first and second exons including the intron of the *DAO2* gene (Figure S2). The gRNAs were designed using a combination of software available online: http://www.rgenome.net/cas-designer/, http://crispr.hzau.edu.cn/cgi-bin/CRISPR2/CRISPR and http://crispor.tefor.net/. The best set of gRNAs was selected based on the efficiency and possible no off-targets. The two gRNAs were assembled into the binary vector pHEE401E using the Golden Gate cloning method as described by [50,51]. *Agrobacterium*-mediated floral dip was used to transform the CRISPR-Cas9 construct into the *dao1-1* background. T1 seedlings were screened on agar media containing 50 μg/mL hygromycin and the surviving seedlings were genotyped for deletions in *DAO2* using specific primers (Table S1). Several T1 transgenic independent lines were found that were either homozygote or heterozygote. The homozygosity for *dao2C* deletion was confirmed in T2. Cas9-construct-free lines were genotyped using specific primers (Table S1). Only homozygote *dao2C* and Cas9 construct-free lines were used for further analysis.

### 4.4. DAO1 Expression Pattern

Seedlings expressing the transcriptional fusion pDAO1:GUS were grown as described above and stained with x-glca cyclohexylammonium salt (Duchefa Biochemie; X1405.1000) as described by [35]. At least 15 seedlings/time point were stained for 2 h and only one representative seedling was photographed.

### 4.5. Gene Expression Experiments

#### 4.5.1. Tissue Preparation

To check the effect of JA and IAA on the expression of *DAO1* and *DAO2*, total RNA was extracted from whole seedlings of wild type and mutants (*coi1-16* or *jin1-2*), which were grown under long-day conditions. Five days after germination, the seedlings were moved to sterile liquid media for overnight acclimation before any treatment. Jasmonic acid (Sigma-Aldrich, J2500, St. louis, MO, USA and IAA (Duchefa Biochemie, I0901) were used in this study. The auxinole was a gift from Professor Hayashi [33].

To check the expression of *AOC2*, *OPR3*, *GH3.3*, *GH3.5*, and *GH3.6* genes, total RNA was extracted from etiolated hypocotyls. Wild-type and *dao1-1* seedlings were first etiolated in the dark until their hypocotyls were 6 mm long (T0), and then they were transferred to long-day conditions for either 9 h (T9), 24 h (T24), or 72 h (T72).

#### 4.5.2. RNA Isolation and cDNA Synthesis

Total RNA was extracted from the prepared plant material using an RNAqueous^®^ Total RNA Isolation kit (Thermo Fisher Scientific Baltics UAB, AM1912, Vilnius, Lithuania). The extracted RNAs were first treated with DNaseI using a DNA*free* Kit (Thermo Fisher Scientific Baltics UAB, AM1906, Vilnius, Lithuania). RNA quantity was checked using a NanoDrop and quality was tested in 1.5% agarose gel (Sigma-Aldrich, J2500, St. louis, MO, USA). cDNA was synthesized by reverse transcribing 1 μg RNA using a SuperScript II Reverse transcriptase kit (Thermo Fisher Scientific, 18064-014) with anchored-oligo (dT)_18_ primer (Thermo Fisher Scientific, SO132) according to the manufacturer’s instructions.

#### 4.5.3. Quantitative RT-PCR (qRT-PCR)

Transcript levels were assessed by qRT-PCR, in assays with triplicate reaction mixtures (final volume, 20 μL) containing 5 μL of cDNA, 0.5 μM of both forward and reverse primers, and 1× LightCycler 480 SYBR Green I Master (Roche, Indianapolis, IN, USA), and quantitative PCR was performed with a LightCycler 480 (Roche) according to the manufacturer’s instructions. A melting curve analysis was added to each PCR program. The sequences of primers used for all target genes are presented in Table S1. The crossing threshold (CT) values for each sample were acquired with the LightCycler 480 software (version number, Roche) using the second derivative maximum method. All quantifications were repeated with at least two independent biological replicates. The relative transcript amount was calculated as described by [34]. Normalization of qRT-PCR was performed using reference gene *TIP41* (Table S1). The data are relative to the calibrator, either mock-treated (in Figure 1) or wild type (in Figure 4E,F).

## 5. Phytohormone Profiling

Seedlings of Col-0 and *dao1-1* were grown under AR phenotyping growth conditions, as described by [34]. Only hypocotyls were collected and rapidly dried on tissue paper then stored in Eppendorf tubes at −80 °C after freezing in liquid nitrogen. Six biological replicates were provided. Endogenous levels of jasmonates (free JA, its conjugates, and intermediates) and auxin metabolites (free IAA, its conjugates IAAsp, IAGlu, and catabolite oxIAA) were quantified from 20 mg fresh weight according to the method described by [52].

## Supplementary Materials

Supplementary materials can be found at https://www.mdpi.com/1422-0067/20/18/4428/s1.
