# Supplementary material for: A DAO1-Mediated Circuit Controls Auxin and Jasmonate Crosstalk Robustness during Adventitious Root Initiation in Arabidopsis
Source: Int J Mol Sci. 2019 Sep 9;20(18):4428. doi: 10.3390/ijms20184428 (PMC6769753; doi:10.3390/ijms20184428)
Supplement: Supplementary file 1 [file ijms-20-04428-s001.pdf]

Supplementary Information

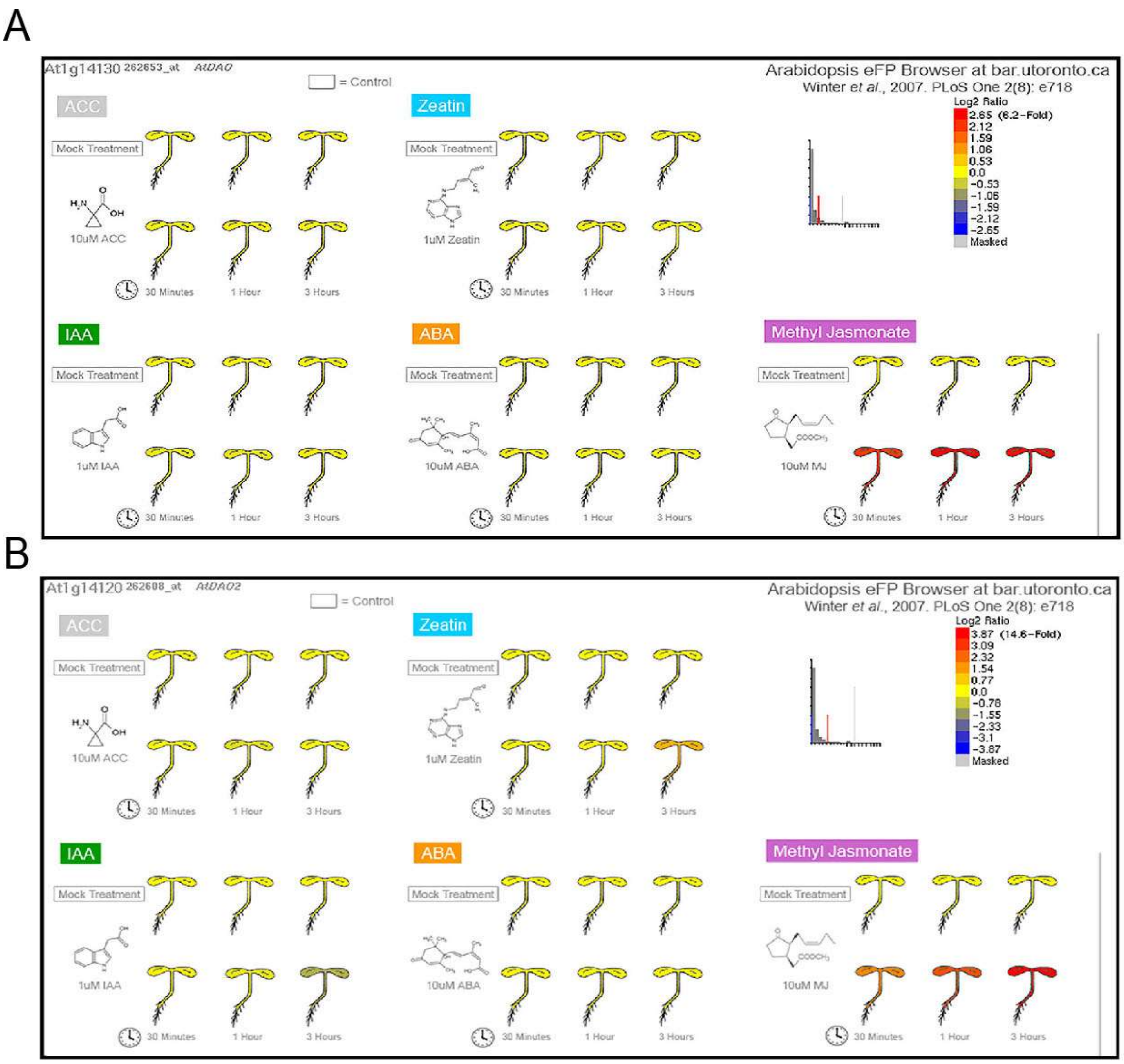

Figure 1. Relative expression data showing that MeJA specifically induces the expression of (A) ***DAO1*** and **(B)** ***DAO2*****.** Data retrieved from the publicly-available dataset at Arabidopsis eFP browser (http://bar.utoronto.ca).

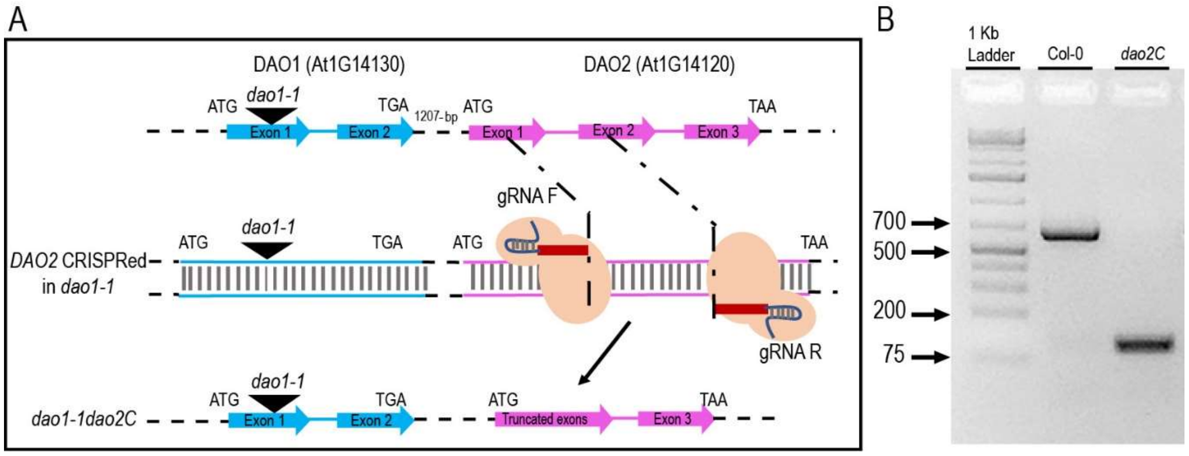

Figure 2. Illustration showing the CRISPR-Cas9 strategy adopted. (A) Two guide RNAs were designed to target a relatively large DNA fragment from the *DAO2* gene in **a** *dao1-1* loss of function mutant background**. (B)** Agarose gel showing 647 bp fragment in the *DAO2* wild type and homozygote deletion of ≈500 bp resulting in ≈100 bp fragment in the *dao2C* allele.

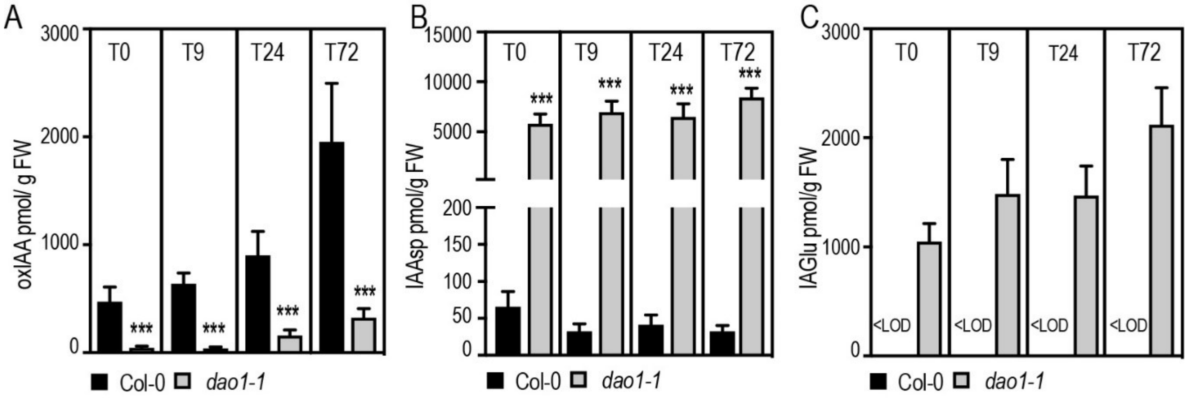

Figure 3. The *dao1-1* mutant accumulates significantly less OxIAA and more amino acid conjugates (IAAsp and IAGlu). **(A-C)** Endogenous hormone contents. **(A)** OxIAA, (B) indole-3-acetyl-L-aspartic acid (IAAsp) and **(C)** indole-3-acetyl glutamic acid (IAGlu) were quantified in the hypocotyls of wild-type and *dao1-1* mutant seedlings grown in the dark until the hypocotyl reached ~ 6 mm long (T0) and after their transfer to the light for 9 h (T9), 24 h (T24) or 72 h (T72). Error bars indicate ± SD of six biological replicates. Asterisks indicate statistically significant difference in the mutant lines versus the wild type (Col-0) in an ANOVA analysis (*, **, and *** correspond to P-values of 0.05 > p > 0.01, 0.01 > p > 0.001, and p < 0.001, respectively). <LOD means under the limit of detection.

Table 1: list of primers used in qRT-PCR and genotyping

Gene name	Gene number	Forward primer	Reverse primer
GH3.3	At1g77850	ACAATTCCGCTCCACAGTTC	ACGAGTTCCTTGCTCTCCAA
GH3.5	At4g27260	GTCTTCGAGGACTGCTGCTT	ATGTCCCTGGCTCAACAATC
GH3.6	At5g54510	CCTTGTTCCGTTTGATGCTT	CGTGTTACCGTTCAAGCAGA
OPR3	At2G06050	TGGTTGGCATGCTCAATAAG	GCCTTCCAGACTCTGTTTGC
AOC2	At3G25770	GGTGCCTACGGACAGGTCAAGC	GCGGTACCGGTGTTCCGGTG
TIP41	At4g34270	GCTCATCGGTACGCTCTTTT	TCCATCAGTCAGAGGCTTCC
dao2C	At1g14120	TTGCTTGACTAGAGAAAAGCCTT	TTGGCTTGGCCATCCTCTCA
U626-ID		TGTCCCAGGATTAGAATGATTAGGC	AGCCCTCTTCTTTCGATCCATCAAC
